# Draft Genome Sequence of Clinical Isolate *Alcaligenaceae* sp. Strain 429

**DOI:** 10.1128/MRA.00439-19

**Published:** 2019-05-09

**Authors:** Michael J. Zilliox, Paul C. Schreckenberger, Catherine Putonti

**Affiliations:** a Department of Public Health Sciences, Loyola University Chicago, Maywood, Illinois, USA; b Department of Pathology, Loyola University Chicago, Maywood, Illinois, USA; c Department of Computer Science, Loyola University Chicago, Chicago, Illinois, USA; d Bioinformatics Program, Loyola University Chicago, Chicago, Illinois, USA; e Department of Biology, Loyola University Chicago, Chicago, Illinois, USA; f Department of Microbiology and Immunology, Loyola University Chicago, Maywood, Illinois, USA; Georgia Institute of Technology

## Abstract

Here, we present the 3.53-Mb genome for *Alcaligenaceae* sp. strain 429, isolated from a patient with unknown etiology. While the 16S rRNA gene most closely resembles *Paenalcaligenes* species, average amino acid identity (AAI) analysis did not meet the threshold to classify our strain as a species of this family.

## ANNOUNCEMENT

The bacterial isolate was received from Elmhurst Hospital in Elmhurst, IL, in 2012 for identification. No patient data are available. Prior to sequencing, identification was performed using a matrix-assisted laser desorption ionization (MALDI) Biotyper (Bruker Scientific, Billerica, MA, USA), which classified the strain as an *Alcaligenes* sp., whereas the Vitek MS system (bioMérieux, Marcy-l’Étoile, France) found no match. However, 16S rRNA gene sequencing performed by our group indicated that the isolate was more closely related to another member of the *Alcaligenaceae* family, Paenalcaligenes suwonensis. *Paenalcaligenes* species have been isolated from both the environment (1) and patients (2, 3), and only one genome has been sequenced for the genus to date (P. hominis). Furthermore, it is rarely associated with clinical symptoms (2, 3). In contrast, infections attributed to Alcaligenes faecalis have been reported, although they are rare and nosocomial (4–8). To resolve the taxonomy of this clinical isolate, we sequenced the genome.

The isolate was originally saved in Brucella broth with 10% glycerol and frozen at –80°C. The frozen sample was then subcultured onto blood agar plates and incubated at 35°C in 5% CO_2_ for 24 to 48 hours. Genomic DNA was extracted from bacterial cells using a validated mixture of lysozyme and mutanolysin and the DNeasy blood and tissue kit (Qiagen, Valencia, CA) (9). All steps were performed in a UV-irradiated, HEPA-filtered PCR workstation to minimize potential contaminants. The library was constructed using the Nextera XT kit (Illumina, San Diego, CA) following the manufacturer’s protocol and sequenced on an Illumina MiSeq platform using a 500-cycle v2 kit (250 × 2 bp) rendering 1,336,945 paired-end reads.

The raw reads first were trimmed using Sickle v1.33 (https://github.com/najoshi/sickle), specifying that trimmed reads of ≤30 nucleotides be removed from consideration (“-l 30”), and then assembled with SPAdes v3.11.1 using the “careful” option (10). Genome coverage was calculated for the contigs using the BBMap tool v37 (http://sourceforge.net/projects/bbmap/). Contigs with a length less than 600 bp or a coverage less than 60 (1 standard deviation from the mean coverage) were removed from further consideration. The coverage was recalculated using BBMap as 98.64×. The remaining 391 contigs were queried via megablast against the nonredundant/nucleotide (nr/nt) database to confirm the genus and species of the isolate; all hits were to sequences of strains belonging to the genera *Paenalcaligenes* and *Alcaligenes*. The contigs have a total length of 3,539,013 nucleotides with a GC content of 50.1% and an *N*_50_ value of 14,837 bp. The genome was annotated using the NCBI Prokaryotic Genome Annotation Pipeline (11). Three rRNA (1 5S, 1 16S, and 1 23S) gene sequences, 48 tRNA gene sequences, and 3,422 protein coding genes were identified.

The 16S rRNA sequence from the assembled genome was compared to NCBI’s 16S rRNA database via blastn, identifying the best hit to the *P. suwonensis* strain ABC02-12 sequence (GenBank accession number NR_133804; query coverage, 93%; identity, 99.16%). Average amino acid identity (AAI), calculated using the AAI-profiler online server (12), identified the nearest neighbor as Alcaligenes faecalis (72%). The number of coding regions shared between our isolate and *Paenalcaligenes* and *Alcaligenes* strains, however, did not exceed 56%. This finding supports recent evidence suggesting that the *Alcaligenes* genus has an open pangenome (13). Thus, at this time, no genus designation can be made.

### Data availability.

The draft whole-genome project for *Alcaligenaceae* sp. strain 429 has been deposited at DDBJ/EMBL/GenBank under accession number SRSN00000000. Raw sequence reads are deposited at DDBJ/EMBL/GenBank under accession number SRR8862657.
